# A comparative study of single nucleotide variant detection performance using three massively parallel sequencing methods

**DOI:** 10.1371/journal.pone.0239850

**Published:** 2020-09-28

**Authors:** Linea Christine Trudsø, Jeppe Dyrberg Andersen, Stine Bøttcher Jacobsen, Sofie Lindgren Christiansen, Clàudia Congost-Teixidor, Marie-Louise Kampmann, Niels Morling

**Affiliations:** 1 Section of Forensic Genetics, Department of Forensic Medicine, Faculty of Health and Medical Sciences, University of Copenhagen, Copenhagen, Denmark; 2 Faculty of Biology, University of Barcelona, Barcelona, Spain; Ohio State University, UNITED STATES

## Abstract

Massively parallel sequencing (MPS) has revolutionised clinical genetics and research within human genetics by enabling the detection of variants in multiple genes in several samples at the same time. Today, multiple approaches for MPS of DNA are available, including targeted gene sequencing (TGS) panels, whole exome sequencing (WES), and whole genome sequencing (WGS). As MPS is becoming an integrated part of the work in genetic laboratories, it is important to investigate the variant detection performance of the various MPS methods. We compared the results of single nucleotide variant (SNV) detection of three MPS methods: WGS, WES, and HaloPlex target enrichment sequencing (HES) using matched DNA of 10 individuals. The detection performance was investigated in 100 genes associated with cardiomyopathies and channelopathies. The results showed that WGS overall performed better than those of WES and HES. WGS had a more uniform and widespread coverage of the investigated regions compared to WES and HES, which both had a right-skewed coverage distribution and difficulties in covering regions and genes with high GC-content. WGS and WES showed roughly the same high sensitivities for detection of SNVs, whereas HES showed a lower sensitivity due to a higher number of false negative results.

## Introduction

Massively parallel sequencing (MPS) has revolutionised clinical genetics and the research within human genetics. MPS has significantly reduced the cost of sequencing per base compared with traditional Sanger sequencing and made it possible to efficiently investigate a large number of genes in several samples at the same time. Multiple approaches for MPS of DNA are available today. These include e.g. targeted gene sequencing (TGS) panels [1–3], whole exome sequencing (WES) [4–7], and whole genome sequencing (WGS) [8–11]. Gene panels involve selective capturing of target regions and are useful when specific genomic regions are analysed, which minimises the chance of incidental findings. Pre-designed panels for purchase have been developed for investigation of regions associated with specific phenotypes and genetic diseases, such as heart diseases [12]. Multiple different inherited heart diseases have been identified, and genetic testing is recommended in heart patients to identify causative variants and to enable possible treatment or preventive measures [13–15]. Genetic testing can subsequently be performed in family members to identify relatives at risk of developing the same disease as the proband. Custom-made gene panels allowing the researcher to design the panel to target regions of interest are also available. An advantage of TGS is the generation of smaller and more manageable datasets compared to WES and WGS. Moreover, by sequencing small parts of the genome, more samples can be sequenced simultaneously with the current technologies, which significantly reduces the costs. As novel gene-disease associations are identified, TGS panels continuously need to be updated to include new gene variants and minimise inconclusiveness in case of negative results. Updates and re-sequencing can be avoided by sequencing a larger proportion of the genome by applying WES or WGS instead. WES sequences the human exome that accounts for approximately 2% of the genome. The WES method involves a selective capturing of target exons. Multiple WES kits are available, and they are not necessarily designed to target all exons, but instead target exons of the most frequent transcripts [16]. In contrast, WGS sequence the entire genome including non-coding regions. With the increase in the understanding of gene regulation and the relationship between non-coding variants and diseases, variants in non-coding regions may be of importance in clinical practice making WGS preferable [8, 17]. However, WGS requires a multitude of sequencing reads of all the genome, which limits the number of samples per sequencing run and increases the costs. Compared to WGS, samples analysed with TGS or WES are typically sequenced to a higher depth. However, since TGS and WES are limited to specific regions, fewer bases are sequenced resulting in a lower cost per sample compared to that of WGS. As WGS generates large datasets, it is also a highly computationally demanding approach. An advantage of WGS is the simple PCR-free library preparation compared to those of TGS and WES protocols with probe capture and PCR steps.

Some advantages and disadvantages exist for the various MPS methods. In many cases, the choice of method is taken mainly considering costs and ethical considerations. TGS panels related to the patients’ phenotypes are often used as the initial test followed by WES if the TGS test is negative, and today WGS is rarely used in clinical practice [18]. An important issue with WES and WGS is the possible incidental findings of disease-related variants in genes irrelevant to the investigated phenotype [19]. Incidental findings may raise ethical difficulties, but can be overcome by in silico restriction of WES and WGS sequencing to predefined regions that are carefully selected for their implications in diseases or phenotypes.

As MPS is an integrated part of the investigations in many genetic laboratories and is increasingly being implemented in clinical and forensic settings, it is of great importance to evaluate the variant detection performance of the various MPS approaches. Former studies have concluded that differences do exist. WGS data has been shown to be of higher quality, more uniform, and with less false variant positives than WES data [20]. A recent study showed that WGS has higher genotype quality, can identify more variants, and is less prone to allelic dropout than WES [21]. Less is known about the performance of TGS panels using different chemistries, including the HaloPlex target enrichment system (HES).

In the present study, we assessed and compared the single nucleotide variant (SNV) detection performances of WGS, WES, and HES using DNA from 10 individuals. The custom-made HES was designed to screen for variations in 100 genes previously shown to be associated with inherited cardiomyopathies and channelopathies [22]. The term SNV was preferred over single nucleotide polymorphism (SNP), because a SNV is not dependent on being present in over 1% of the population. This allow the identification of rare variants that are often associated with inherited diseases, e.g. inherited cardiac diseases.

## Materials and methods

### Ethics

The study was approved by the Committees on Health Research Ethics in the Capital Region of Denmark (H-2-2012-017) and the Danish Data Protection Agency (2011-54-1262).

### DNA extraction

DNA was purified from whole blood of 10 deceased individuals autopsied at the Department of Forensic Medicine, Faculty of Health and Medical Sciences, University of Copenhagen, Denmark in the period 2009–2011. Extraction was performed using the QIAamp DNA Blood Mini kit (Qiagen, Germany) following the manufacturer’s recommendations. The DNA concentration was assessed using a Qubit fluorometer 2.0 with the dsDNA HS assay (Invitrogen, USA).

### Library preparation and sequencing

#### Target gene sequencing using the HaloPlex Target Enrichment System

Exons with 25 base pairs (bases) of the adjacent introns and the 5´- and 3´-UTR of 100 cardiac channelopathies and cardiomyopathies [22] were isolated and captured using a custom design of the HaloPlex Target Enrichment System (HES). The HaloPlex PCR Target Enrichment protocol version D.5 (Agilent Technologies, USA) was used for library preparation according to the manufacturer’s instructions. Libraries were quantified using a Qubit fluorometer 2.0 with the dsDNA HS assay (Invitrogen, USA). The library size distribution was analysed using a 2100 Bioanalyzer and the High Sensitivity DNA kit (Agilent Technologies, USA). All DNA samples were sequenced with the MiSeq platform (Illumina, USA) with paired-end sequencing (2x150 bases) using the MiSeq Reagent Kit V2 (300 cycles) following the manufacturer’s recommendations.

#### Whole exome sequencing

Prior to WES and WGS library preparation, fragmentation of DNA to insert size 350 bases was performed with ultrasonication using a Covaris S220 instrument and the SonoLab 7.1 software (Covaris Inc, USA). The libraries were quantified using a Qubit fluorometer 2.0 with the dsDNA HS assay (Invitrogen, USA). The library size distribution was analysed using a 2100 Bioanalyzer and the High Sensitivity DNA kit (Agilent Technologies, USA).

The SureSelect XT Target Enrichment System for Illumina Paired-end Sequencing version B4 (Agilent Technologies, USA) was used to prepare exomes for sequencing, and the SureSelect Clinical Research Exome (CRE)—Library version 1 (Agilent Technologies, USA) was used to capture the human exome.

Samples were sequenced with the NextSeq500 platform (Illumina, USA) using paired-end sequencing (2x150 bases).

#### Whole genome sequencing

Library preparation for WGS was performed with the TruSeq^®^ DNA PCR-Free Library Prep kit (Illumina, USA) following the manufacturer’s recommendations (Revision D June 2015) with the modification that the NEBNext End Repair Module (New England Biolabs, USA) was used for end-repair according to the manufacturer’s instructions.

Samples were sequenced with the NextSeq500 platform (Illumina, USA) using paired-end sequencing (2x150 bases).

### Variant detection

#### HaloPlex variant detection using SureCall

SureCall version 3.0.3.1 (Agilent Technologies, USA) was used for variant detection of the HaloPlex Target Enrichment System. In brief, FASTQ files were used as input followed by adapter removal and read alignment to the UCSC human genome version 19 (hg19/GRCh37), released Feb 2009, using Burrows-Wheeler Aligner (*BWA)-MEM* version 0.7.10 [23]. The BAQ SNP caller using *SAMtools* [24] was used to perform local realignment, indexing, and variant calling. Lists of identified variants were created in variant call format (VCF) [25]. Finally, QC reports were generated providing quality metrics for each sample.

The SureCall default settings of the minimum read depth was changed from 40 to zero. Detailed SureCall analysis parameters are provided in S1 Table.

#### Whole exome and whole genome variant detection

The NextSeq500 output base call (BCL) files were converted to FASTQ files using *bcl2fastq* (Illumina, USA). *AdapterRemoval* [26] version 2.1.3 identified and removed adapter sequences from the reads using the collapsed option. Consecutive stretches of low-quality bases (Q<30) were removed from the 5’ and 3’ termini, and reads shorter than 30 bases were discarded. The Phred+33 quality scores encoding was used. Reads were aligned to hg19 using *BWA-MEM* version 0.7.10 with default parameters [23]. Only properly aligned reads (*samtools* flag–f 0x2) were accepted. The resulting Sequence Alignment/MAP (SAM) files were converted into binary alignment map (BAM) files using *SAMtools* version 1.0 [24]. The genome analysis toolkit (*GATK*) version 3.2.2 and *HaplotypeCaller* [27] were used with default settings for variant calling (S1 Table). Lists of variants were provided in VCF.

### Comparison of single nucleotide variation detection performances

The comparison of SNV detection performances among the three methods was carried out as a two-step investigation: 1) Pairwise comparison between the three methods, WGS and WES, WGS and HES, and WES and HES. This was carried out to investigate the maximum number of shared bases between two methods, since all three methods did not sequence the same regions. 2) Comparison of the SNV detection performance in the regions sequenced by all three methods. Information about captured regions for WES and HES was obtained through Agilent Technologies, USA, and WGS was expected to cover all investigated regions. Comparison of the detection performance was carried out in R (R core team, version 3.5.0, http://www.R-project.org/).

#### Pairwise comparison between methods and identification of fully exclusive and high quality fully exclusive variants

The three pairwise comparisons were restricted to regions covered by the methods compared. When comparing WGS and WES, the comparison was restricted to WES captured regions of the 100 cardiac genes (600,279 bases) included in the HES panel described by Hertz et al. [22]. WGS and HES was restricted to HES captured regions (783,503 bases), and 432,075 bases was found in the overlapping captured regions between WES and HES. All three methods covered the 432,075 bases shared between WES and HES.

For the pairwise comparisons, we separated variants into two categories: 1) SNVs identified by one method but not by the other were referred to as fully exclusive (FE) variants (no quality filtering criteria were required for a SNV to be identified as a FE), and 2) FE variants that passed variant quality filtering criteria were referred to as high-quality FE variants (HQFE). HQFE variants were identified as FE variants with a minimum read depth (10x for WGS and 40x for both WES and HES), and a balanced heterozygous allele balance (AB—minor allele / total number of alleles between 0.2 and 0.5).

All FE variant loci were examined with Integrative Genomics Viewer (IGV) [28]. The positions of the FE variants were investigated in the alignment file (BAM-file).

#### Comparison of the SNV detection performance in the regions sequenced by all three methods

**Fig 1:**
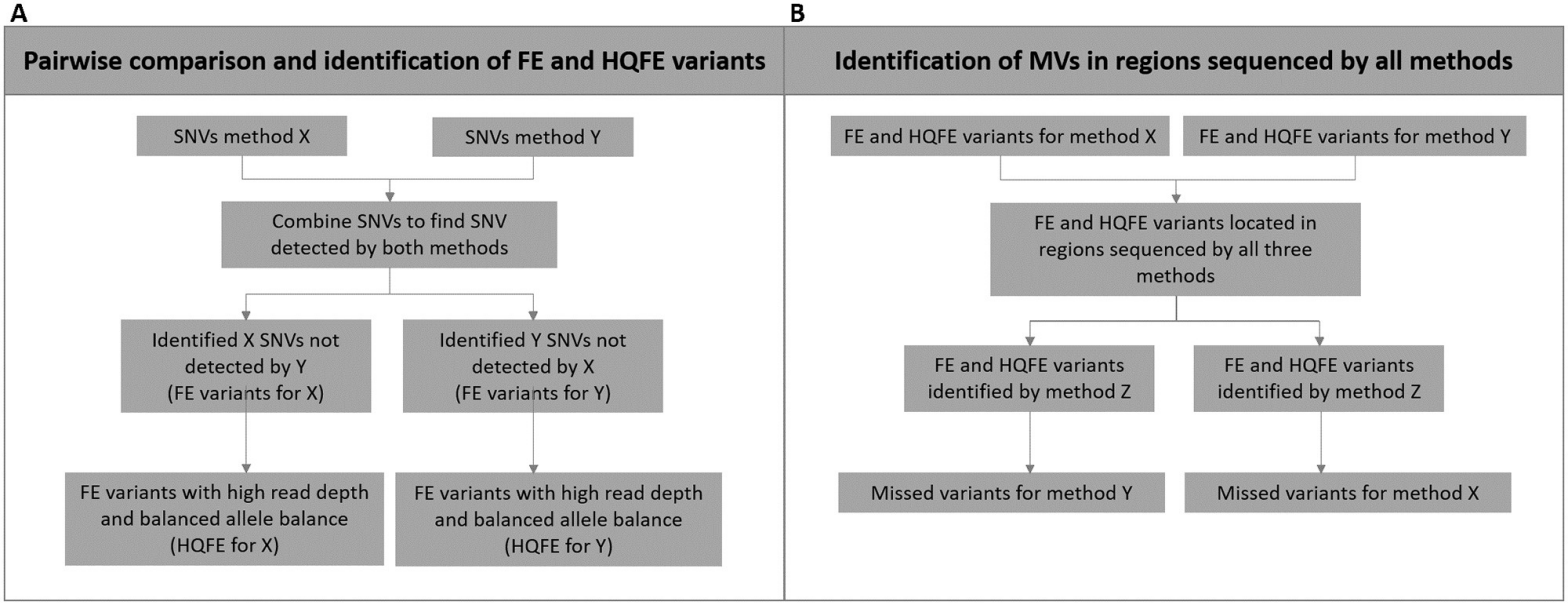
Flow diagram for the comparison of single nucleotide variant (SNV) detection performances of whole genome sequencing (WGS), whole exome sequencing (WES), and HaloPlex target enrichment sequencing (HES). A: Pairwise comparisons. For each sample, SNVs from methods X and Y were compared. Fully exclusive (FE) variants were identified as SNVs that were only detected by one of the two methods compared. FE variants with high read depth and balanced allele balance were defined as high quality fully exclusive (HQFE) variants. B: Identification of missed variants (MVs). FE and HQFE variants for methods X and Y in regions sequenced by all methods were compared to those of the third method not used in the pairwise comparison (method Z). Method X FE and HQFE variants also identified by method Z were identified as MVs for Y, and method Y FE and HQFE variants also identified by method Z were identified as MVs for X.

The pairwise detected FE and HQFE variants located in regions sequenced by all three methods were hereafter investigated with all three methods to identify if a method failed to detect variants that was detected by the two other methods (missed variants—MVs). See Fig 1 for a flow diagram of the two-step investigation of the SNVs.

## Results

The SNV detection performances of three MPS methods (WGS—Illumina TruSeq^®^ DNA PCR-Free Library Prep kit, WES—Agilent SureSelect Clinical Research Exome, and HES–Agilent HaloPlex Target Enrichment System) were investigated in 100 cardiac gene regions in 10 individuals.

With the three methods, different regions of the 100 cardiac genes were investigated. The WGS methods is a PCR-free sequencing of the whole genome and, therefore, sequences both exonic, intronic, and intergenic regions, whereas WES and HES use capture probes to selected the regions of the DNA to be sequenced. The WES method captures exons of the most frequent transcripts of genes throughout the genome, whereas the custom-designed HES captures all exons and short parts of the flanking regions of the 100 cardiac genes as described by Hertz et al. [22]. Because the three methods investigated different regions of the 100 cardiac genes, the comparison of SNV detection performances was carried out in a two-step investigation: 1) Pairwise comparison between the three methods, WGS and WES (600,279 bases), WGS and HES (783,503 bases), and WES and HES (432,075 bases), and 2) Comparison of the SNV detection performance in the regions sequenced by all three methods (432,075 bases).

### Coverage

**Fig 2:**
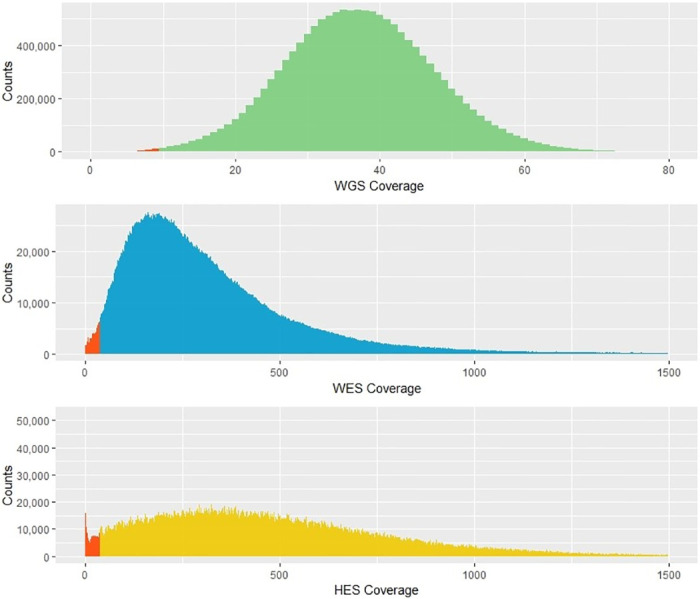
Coverage distribution of the three sequencing methods. The green histogram shows the coverage distribution for whole genome sequencing (WGS), the blue histogram shows the coverage distribution for whole exome sequencing (WES), and the yellow histogram shows the distribution for HaloPlex Enrichment sequencing (HES). For all three histograms, the red bars show counts of low-covered bases (<10x for WGS and <40x for WES and HES).

The coverages of the three methods were compared (Fig 2). WGS had a normally distributed coverage with mean 37 and median 37. WES and HES had right-skewed (positive) distributions with mean 332 and median 265 for WES, and mean 482 and median 431 for HES. WGS covered the largest proportion of the investigated regions compared to WES and HES. However, all methods covered >99% of the investigated bases (S2 Table). For each method, we defined low-covered bases. For WGS, low-covered bases were defined as less than 10x coverage, and for WES and HES less than 40x coverage. On average, WGS had 0.3% low-covered bases compared to 1.7% low-covered bases with WES and 3.3% low-covered bases with HES.

### Coverage per gene

**Fig 3:**
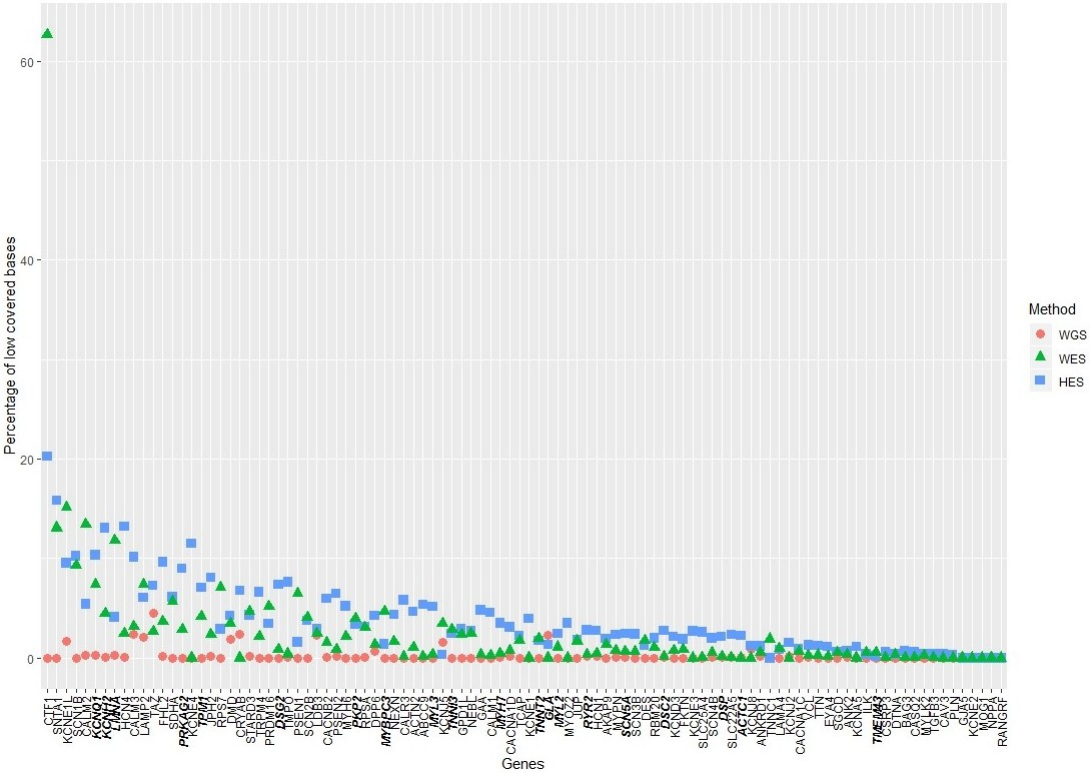
Percentage of low-covered bases per gene for each method. The genes were ordered by the percentage of low-covered bases. The red dots represent whole genome sequencing (WGS), the green triangles represent whole exome sequencing (WES), and the blue squares represent HaloPlex target enrichment sequencing (HES). Gene names in both bold and italic are found in the ACMG SF v.2.0 list of genes published by the American College of Medical Genetics and Genomics (ACMG).

From a clinical point of view, it is important to cover interesting genes with high coverage and avoid bases or sequences with low coverage. We assessed the coverage per gene by investigating the percentage of low-covered bases per gene (Fig 3). WGS covered all 100 cardiac genes with high coverage per gene (≥10x). HES had 10 genes with more than 10% low-covered bases (<40x), and WES had five genes with more than 10% low-covered bases (<40x). Common to WES and HES was the high percentage of low-covered bases in *CTF1* (20% for HES and 63% for WES), *SNTA1* (16% for HES and 13% for WES), *KCNE1L* (10% for HES and 15% for WES), and *SCN1B* (10% for HES and WES). For these genes, WGS had <1% low-covered bases. The American College of Medical Genetics and Genomics (ACMG) has published recommendations for reporting incidental findings in the exons of certain genes (ACMG SF v.2.0) [29]. The list comprises 66 genes and in this study we investigated 20 as part of the 100 cardiac genes. The 20 investigated ACMG SF v.2.0 listed genes (*KNCNQ1*, *KCNH2*, *LMNA*, *PRKAG2*, *TPM1*, *DSG2*, *PKP2*, *MYBPC3*, *MYL3*, *TNNI3*, *MYH7*, *TNNT2*, *GLA*, *MYL2*, *RYR2*, *SCN5A*, *DSC2*, *DSP*, *ACTC1*, and *TMEM43*) are shown both in bold and italic in Fig 3. WGS covered all investigated ACMG SF v.2.0 genes with high coverage, whereas HES and WES had genes with more than 5% low-covered bases (HES: *KCNQ1*, *KCNH2*, *LMNA*, *PRKAG2*, *TPM1* and *DSG2*. *WES*: *KCNQ1* and *LMNA*).

### Discordant single nucleotide variants in the pairwise comparisons

We investigated the number of discordant SNV loci among the three different sequencing methods (Table 1). Pairwise comparisons between the three methods (WGS vs. WES, WGS vs. HES, and WES vs. HES) were carried out. SNVs called exclusively by one method in the pairwise comparison were separated into two categories, fully exclusive (FE) and high quality FE (HQFE) variants. FE variants were defined as variants found by one method and not by the other method. HQFE variants were defined as FE variants that passed the quality filtering criteria of min coverage (10x for WGS and 40x for WES and HES) and heterozygous allele balance (AB—minor allele / major allele from 0.2 to 0.5). The median number of FE and HQFE variants per sample in the three comparison experiments are shown in Table 1. To find method-specific characteristics for the FE variants, all detected FE variants were inspected using IGV (S3–S5 Tables). HES showed most FE variants in both the pairwise comparison with WGS and WES (S4 and S5 Tables). In addition, HES had the largest number of FE variants in repetitive regions. If the repetitive regions were excluded, WGS showed more FE variants compared to HES (S4 Table).

**Table 1:** Number of fully exclusive (FE) and high quality FE (HQFE) single nucleotide variants (SNVs) in the three comparisons.

Comparison	Median no. per sample	
WGS and WES	WGS	WES
SNVs	402	395
FE	6 (1.5%)	1 (0.3%)
HQFE	3 (0.7%)	1 (0.3%)
WES and HES	WES	HES
SNVs	225	226
FE	2 (0.9%)	3 (1.3%)
HQFE	1 (0.4%)	1 (0.4%)
WGS and HES	WGS	HES
SNVs	628	636
FE	19 (3%)	32 (5%)
HQFE	15 (2.4%)	17 (2.7%)

The percentage FE or HQFE variants of SNVs is shown in parentage.

### Fully exclusive variants and missed variants in regions sequenced by all three methods

**Fig 4:**
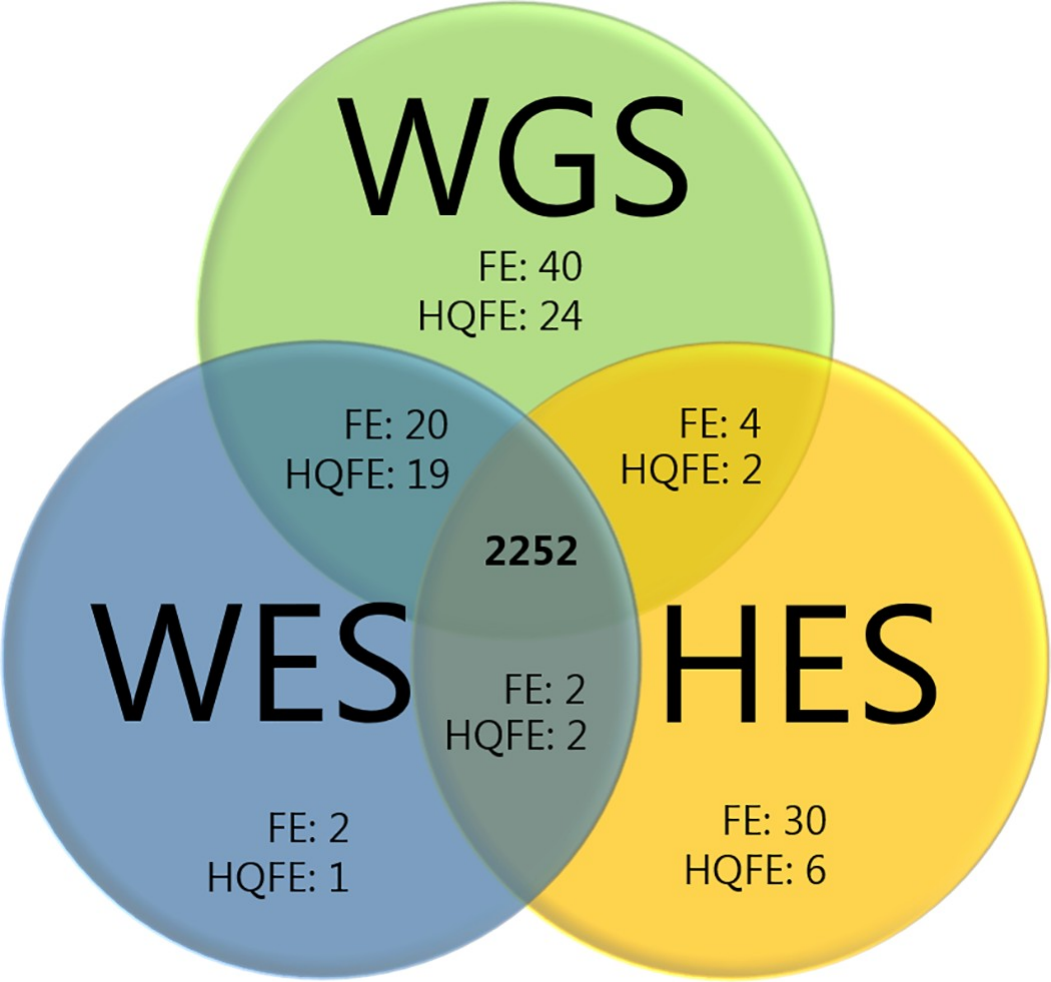
Venn diagram of overlapping single nucleotide variants (SNVs) in regions sequenced by all methods. WGS: whole genome sequencing, WES: whole exome sequencing, HES: HaloPlex target enrichment sequencing, FE: fully exclusive variants, and HQFE: high quality fully exclusive variants.

To further investigate the pairwise detected FE variants, we compared the FE variants in regions sequenced by all three methods (Table 2 and Fig 4). For example, if a WGS FE variant in the comparison with WES was also found to be a HES FE in the comparison with WES, it was confirmed as a FE variant and a missed variant (MV) of WES. WGS detected 64, WES 24, and HES 36 FE variants in the regions sequenced by all methods. Of these, WGS had 24 out of 64 variants confirmed by another method, WES 22 out of 24, and HES six out of 36. To evaluate the ability to detect a variant, we calculated the true positive rate (sensitivity) for each method. The true positive rate was calculated as the proportion of confirmed variants out of the total number of true positives (sum of confirmed variants and MVs). WGS and WES showed very high sensitivities (99.9% for WGS and 99.8% for WES), whereas HES had a lower sensitivity of 99.1%. The lower sensitivity for HES was a result of the higher number of MVs (false negatives) compared to those of WGS and WES.

**Table 2:** Fully exclusive (FE) and high quality fully exclusive (HQFE) variants in regions sequenced by all methods.

		WGS	WES	HES
Total no. of detected SNVs		2316	2276	2288
SNVs confirmed by all methods		2252	2252	2252
FE		64	24	36
	Confirmed	24	22	6
	Not confirmed	40	2	30
	Missed variants (MVs)	2	4	20
	True positive rate (sensitivity)	99.9%	99.8%	99.1%
HQFE		45	22	10
	Confirmed	21	21	4
	Not confirmed	24	1	6
	MVs	2	2	19
	True positive rate (sensitivity)	99.9%	99.9%	99.1%

Both WGS and HES had many FE variants that were not detected by any other method. This may indicate that WGS and HES had higher false discovery rates compared to WES. If quality filtering criteria (min coverage and heterozygous allele balance–HQFE variants) were applied to the WGS and HES FE variants, the number of variants dropped considerably. The number of non-confirmed WGS FE variants was decreased from 40 to 24 non-confirmed HQFE variants, and the number of HES FE variants were decreased from 30 to six HQFE, supporting that 16 of the WGS FE variants and 24 of the HES FE variants were false positives. The sensitivity after quality filtering criteria (HQFE) was 99.9% for both WGS and WES sensitivities of 99.9%, while HES had a sensitivity of 99.1%.

### Fully exclusive variants and missed variants per gene in regions sequenced by all methods

**Fig 5:**
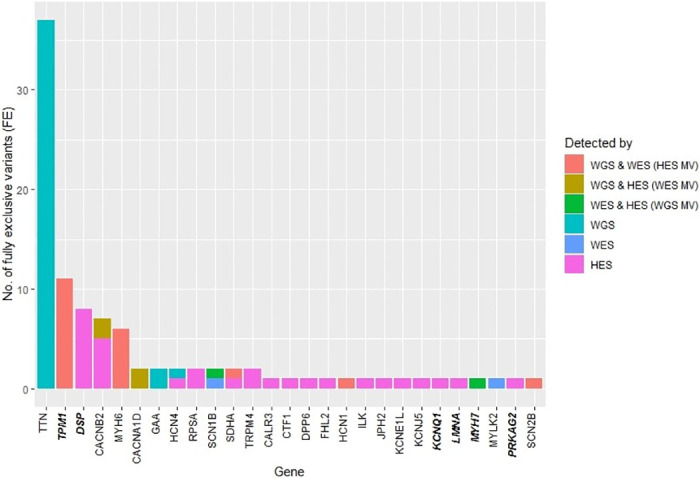
Barplots of fully exclusive (FE) variants per gene. The colours denote the methods that detected the variants. The FE variants were found within regions sequenced by all methods. Gene names in both italic and bold were found in the ACMG SF v.2.0 list of genes published by the American College of Medical Genetics and Genomics (ACMG). The genes were ordered according to the numbers of FE variants. WGS: Whole genome sequencing, WES: Whole exome sequencing, HES: HaloPlex target enrichment sequencing, MV: Missed variant.

**Fig 6:**
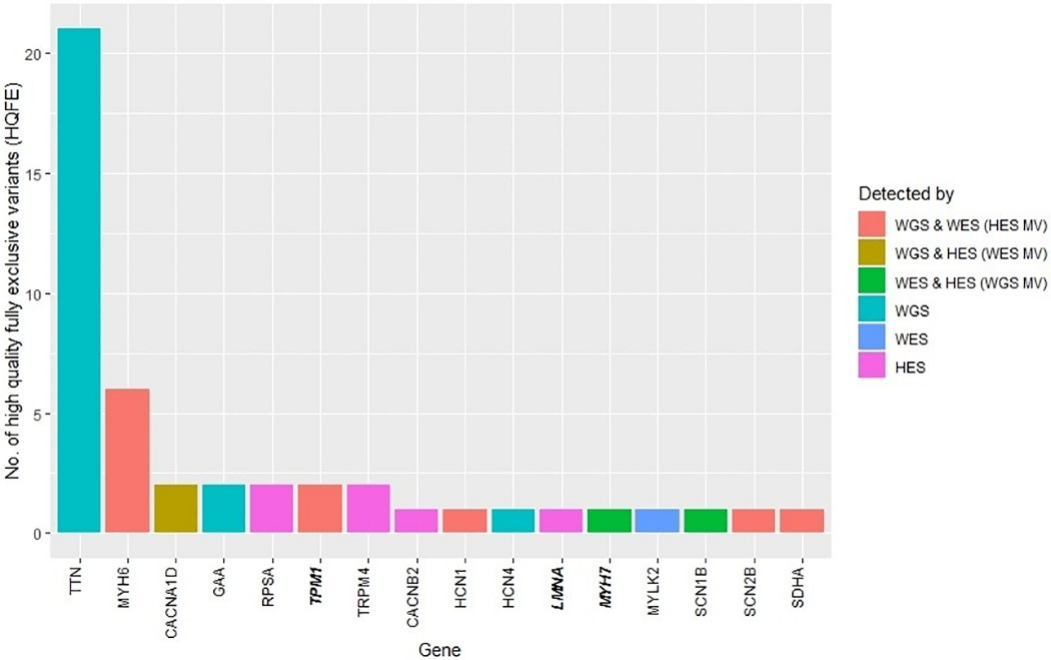
Barplots of high quality fully exclusive (HQFE) variants per gene. The colours denote the methods that detected the variants. The HQFE variants were found within regions sequenced with all methods. Gene names in both italic and bold were found in the ACMG SF v.2.0 list of genes published by the American College of Medical Genetics and Genomics (ACMG). The genes were ordered according to the numbers of HQFE variants. WGS: Whole genome sequencing, WES: Whole exome sequencing, HES: HaloPlex target enrichment sequencing, MV: Missed variant.

The number of FE variants per investigated cardiac gene was examined (Fig 5). Of the 20 HES MVs, 11 were found in *TPM1*, six in *MYH6*, one in *SDHA*, one in *HCN1*, and one in *SCN2B*. WES had four MVs in *CACNB2* and in *CACNA1D*. WGS had two MVs that were found in *SCN1B* and *MYH7*. WGS had 40 FE variants with *TTN* harbouring 37 variants, two were found in *GAA*, and one in *HCN4*. Of the 37 WGS FE variants, 21 were HQFE found in *TTN* (Fig 6). HES had 30 FE variants in 17 different genes. Of these, only six of the variants were HQFE found in *RPSA* and *TRPM4*. WES had two FE variants located in *SCN1B* and *MYLK2*, but only the variant in *MYLK2* was a HQFE variant.

### Discordant genotype annotation in detected single nucleotide variants

We examined the genotype annotations of the detected SNVs and compared them among the three methods (S6 Table). The methods may identify the same locus as having a SNV, but may annotate the genotype of the SNV differently. In the regions sequenced with all methods, the genotypes of five SNVs differed among the three methods. In four out of the five loci, WES and WGS resulted in the same genotypes (all heterozygous), whereas HES detected the SNVs as homozygous for the variant allele. In the fifth locus, WGS and HES detected a heterozygous genotype and WES detected a homozygous genotype.

### Read depth and allele balance in the regions sequenced by all methods

**Fig 7:**
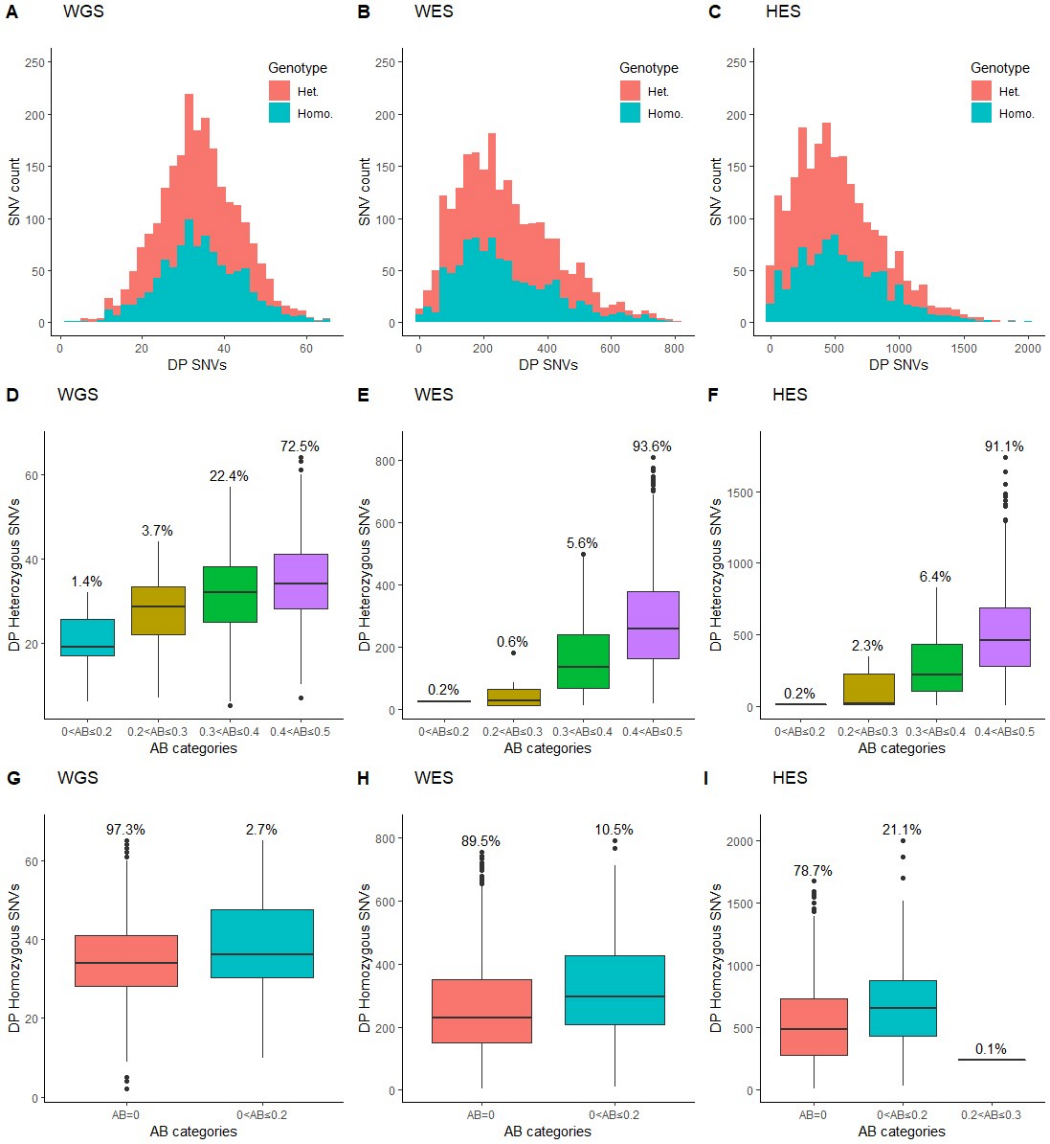
Read depth and allele balance plots. A-C: Read depth (DP) distribution for SNVs detected by whole genome sequencing (WGS), whole exome sequencing (WES), and HaloPlex target enrichment sequencing (HES). D-F: Boxplots showing DP for heterozygous SNVs within each allele balance (AB—minor allele / total number of alleles) category for WGS, WES, and HES. Mean percentage of heterozygous SNVs within each AB category is shown above each boxplot. G-I: Boxplots showing DP for homozygous SNVs within each AB category for WGS, WES, and HES. Mean percentage of homozygous SNVs within each AB category is shown above each boxplot.

We analysed the method specific read depth distribution for SNVs within the regions sequenced by all methods (Fig 7). The allele balance (AB—minor allele / total number of alleles) and the read depth was investigated separately for heterozygous and homozygous SNVs. For heterozygous SNVs, all methods had the highest percentage of SNVs with AB between 0.4 and 0.5 and the lowest percentage of SNVs with unbalanced AB (AB of 0–0.2). A statistically significant (p<0.05) increase in DP with increase in AB was observed for all methods.

The homozygous SNVs were expected to have AB of 0. However, a total of 2.7% WGS annotated homozygous SNVs were found to have minor allele counts, whereas WES had 10.5% and HES had 21.2% annotated homozygous SNVs with minor allele counts. Annotated homozygous SNVs with minor allele counts were found to have statistically significantly (p<0.05) higher read depths than homozygous SNV without minor allele counts for all three methods.

## Discussion

Both in the clinical and forensic settings, genetic investigation using MPS has proven a valuable tool to identify genetic variants [6]. In this study, we aimed to assess and compare the SNV detection performance of the three MPS methods: WGS, WES, and HES. From a clinical application point of view, it is important to obtain high coverages of the nucleotides of the investigated genes. Low-covered regions within functionally important genes could affect variant discovery and clinical diagnosis. We found that WGS covered the largest proportion of the investigated cardiac gene regions (S2 Table). As expected due to the PCR-free workflow, WGS resulted in normally distributed coverages, whereas PCR amplifications with WES and HES protocols resulted in right skewed coverage distributions (Fig 2). Even though all methods were able to cover >99% of the investigated regions, they differed in the average percentage of low-covered bases. WGS performed better than WES and HES, and the highest percentage (3.3%) of low-covered bases was observed for HES (S2 Table). This finding was also reflected in the number of genes with more than 10% low-covered bases (WGS: zero, WES: five, and HES: 10) (Fig 3). Common to WES and HES, we observed four genes (*CTF1*, *SNTA1*, *KCNE1L*, and *SCN1B*) of the 100 investigated genes with more than 10% low-covered bases. The ACMG working group recommends that regions of diminished or absent coverage in the genes examined for incidental findings should supplemented with other investigations [30]. Both the WES and HES methods had ACMG listed genes with more than 5% low-covered bases (HES: *KCNH2*, *KCNQ1*, *LMNA*, *PRKAG2*, *DSG2*, and *TPM1*. *WES*: *LMNA* and *KCNQ1*). Sequencing to higher coverage most likely would decrease the number of low-covered bases in these genes, but would lead to increased cost per investigated sample. The incomplete coverage, as observed in HES and WES, can result from poor enrichment of especially GC-rich and repetitive regions as well as the absence of capture probes for certain regions. A marked reduction in coverage of highly GC-rich regions (>60%) has been shown for methods utilising capture probes, including the Agilent SureSelect kit [31, 32], and low GC-content has likewise been shown to result in decreased coverage when capture-probes were used [32]. We calculated the GC-content of the coding sequences (CDS) of *CTF1*, *SNTA1*, *KCNE1L*, and *SCN1B*, and found that *CTF1*, *SNTA1*, *KCNE1L*, and *SCN1B* had GC-contents of 73%, 64%, 69%, and 56%, respectively, which were above the average of the CDS of ~18,000 widespread RefSeq genes human genes [33]. These findings suggest that high GC-contents could be the reason for the low coverage with WES and HES of these genes. We found that 18% of the HQFE WGS variants were located within GC-rich regions, whereas only 5% of the HQFE HES variants were located in GC-rich regions (S4 Table). This could indicate that WGS performs better than HES for SNV detection in GC-rich regions. In addition, WES was also found to perform better than HES in detecting SNVs in GC-rich regions (S5 Table).

Previous studies have suggested that the qualities of DNA sequencing with WGS and WES are similar [34], and that WES is an efficient alternative to WGS [31, 35]. In support of these findings, we did not observe much difference in the median number of HQFE variants for WES and WGS in the pairwise comparison (Table 1). In the regions sequenced with all methods, both WES and WGS had very high sensitivities for detection of FE and HQFE, whereas HES had a lower sensitivity (Table 2). The difference was caused by the higher number of MVs (false negatives) for HES compared to WGS and WES. Therefore, our results suggest that HES has a greater risk of failing to detect SNVs compared to WGS and WES. WGS and HES showed more variants that were not confirmed by any other method. This indicates that WGS and HES may have a higher false discovery rate compared to WES. Especially, the number of HES FE variants dropped markedly if quality filtering criteria were applied and was decreased from 30 FE variants to six HQFE variants (Table 2). The number of 40 WGS FE variants was decreased to 16 HQFE variants when quality filtering criteria were applied. However, some of the HQFE variants may also be caused by poor SNV detection of the other methods that were needed to confirm the variant. Therefore, it is difficult to conclude that these variants were false positives.

From a clinical point of view, false positives and especially MVs (false negatives) could lead to incorrect conclusions with potentially severe consequences. Another factor that could be clinically important and potentially lead to wrong conclusions is the genotype annotation of detected SNVs. We did identify SNVs with discordant genotype annotation among the three methods. HES accounted for the majority of genotype differences. All HES SNVs were genotyped as homozygous, whereas WES and WGS detected the SNVs as heterozygous. Capture probes might have a higher binding affinity for one of the alleles in heterozygotes and, thereby tend to preferentially capture alleles. Likewise, PCR can introduce bias by amplifying one allele more efficiently than the other [36]. As observed for the HES SNVs with discordant genotypes, a biased detected of one of the alleles could ultimately lead to a heterozygous SNV being wrongly annotated as a homozygous SNV. The use of capture probes and PCR was also expected to result in increased allele imbalance compared to capture- and PCR free SNV detection. Contradictory to this hypothesis, we found that WGS had the lowest frequency (72.5%) of balanced heterozygous SNVs (AB of 0.4≥0.5) compared with WES (93.6%) and HES (91.1%). However, this may be due to the lower overall coverage of WGS compared with WES and HES. In support of this, we found statistically significant correlations (p<0.05) between heterozygote imbalance and low read depth with all methods (Fig 7). We also found annotated homozygous SNVs with minor allele counts. Homozygous SNVs with minor allele counts had statistically significantly (p<0.05) higher read depths with all methods.

This study has some limitations. One of the major limitations to this study is the bioinformatic pipeline used to call the variants (S1 Table). The WES and WGS pipelines employed were the same, whereas HES data was analysed using the Agilent SureCall software. We applied the default SureCall settings, but we acknowledge that differences in pipelines influence the calling of variants and account for some of the observed differences. Due to the special biochemical library preparation design of HES, it was not possible to apply the same variant calling pipeline as for WES and WGS. In addition, we have defined low-covered bases differently for the three methods (<10x for WGS and <40x for WES and HES). The method specific thresholds for minimum read depth was also used in the quality filtering criteria for identifying a variant as HQFE, however, the AB threshold were the same for all three methods. This influences the number of detected HQFE variants. For this reason, we have also chosen to show the FE variants that are the identified variants without quality filtering criteria.

To conclude, our results highlight WGS as the best method for SNV detection when it comes to sensitivity and coverage distribution. However, we also found WES to have a similar high sensitivity for SNV detection, but WES was less efficient due to insufficient coverage of specific regions covering *CTF1*, *SNTA1*, *KCNE1L*, *SCN1B*, and the ACMG listed genes *LMNA* and *KCNQ1*, most likely due to the high GC-contents of these regions. Our findings also propose that HES performs poorer than WES and WGS when it comes to both SNV detection and coverage in the investigated regions. The results also indicate that sequencing to a higher read depth would result in lower proportions of unbalanced heterozygous SNVs for all three methods, but also increase the proportion of annotated homozygous SNVs with minor allele counts.

## Supporting information

S1 Table Variant calling pipeline for whole genome sequencing (WGS), whole exome sequencing (WES), HaloPlex target enrichment sequencing (HES). (DOCX)

S2 Table Coverage characteristics of the comparison among the three methods. WGS: Whole genome sequencing, WES: Whole exome sequencing, and HES: HaloPlex target enrichment sequencing.
(DOCX)

S3 Table Fully exclusive (FE) and high quality fully exclusive (HQFE) variants from whole genome sequencing (WGS) and whole exome sequencing (WES) comparison. (DOCX)

S4 Table Fully exclusive (FE) and high quality fully exclusive (HQFE) variants from whole genome sequencing (WGS) and target enrichment sequencing (HES) comparison. (DOCX)

S5 Table Fully exclusive (FE) and high quality fully exclusive (HQFE) variants from whole exome sequencing (WES) and target enrichment sequencing (HES) comparison. (DOCX)

S6 Table Single nucleotide variants (SNVs) with discordant genotypes. WGS: Whole genome sequencing, WES: whole exome sequencing, and HES: Haloplex target enrichment system. 1/1 shows a homozygote variant genotype and 0/1 shows a heterozygote genotype. SNVs in italic are found in the regions sequenced by all methods.
(DOCX)
