# Supplementary material for: A comparative study of single nucleotide variant detection performance using three massively parallel sequencing methods
Source: PLoS One. 2020 Sep 28;15(9):e0239850. doi: 10.1371/journal.pone.0239850 (PMC7521702; doi:10.1371/journal.pone.0239850)
Supplement: S1 Table — (DOCX) [file pone.0239850.s001.docx]

**S1 Table. Variant calling pipeline for Whole genome sequencing (WGS), Whole exome sequencing (WES), HaloPlex target enrichment sequencing (HES).**

| **SureCall parameters for analysis of HES data** | **In-house variant calling pipeline for WES and WGS** |
| --- | --- |
| **Adapter removal, trimming**   - Trim bases from end of reads if quality of bases are less than 5 - If read is trimmed to a length <30% of original read length, the reads is discarded - Alignment using *BWA-MEM* | - AdapterRemoval  (min trim length: 30, min trim quality: 30) - Map paired-end files using *BWA-MEM* v. 0.7.10 - SAM to BAM - Statistics mapped reads - Extract only the proper paired mapped reads (-f 0x2) - Statistics mapped reads after extraction - Mark duplicates (do not remove) - Merge BAM if more exists - Sort BAM, index BAM - Re-alignment of reads around indels - Write out re-calibrated BAM - GATK, HaplotypeCaller v. 3-6 using the collapsed function |
| **Post alignment processing**   - Do not remove duplicates |  |
| **Variant detection, BAQ SNP caller (*SAMtools*)**   - If no mate read the read is discarded - Min 13 base quality to be considered a mutation - Min 0 read quality to be used for variant detection - Minimum average distance of variant from 3’ end = 5. From 5’ end = 0 - Max MAPQ difference between variant and WT = 20 - Read depth SNVs: 0 (default 40) |  |
