# Supplementary material for: A comparative study of single nucleotide variant detection performance using three massively parallel sequencing methods
Source: PLoS One. 2020 Sep 28;15(9):e0239850. doi: 10.1371/journal.pone.0239850 (PMC7521702; doi:10.1371/journal.pone.0239850)
Supplement: S2 Table — WGS: Whole genome sequencing, WES: Whole exome sequencing, and HES: HaloPlex target enrichment sequencing. (DOCX) [file pone.0239850.s002.docx]

**S2 Table. Coverage characteristics of the comparison among the three methods.** WGS: Whole genome sequencing, WES: Whole exome sequencing, and HES: HaloPlex target enrichment sequencing.

| **Comparison** | **WGS** | **WES** | **HES** |
| --- | --- | --- | --- |
| **WGS and WES (600,279bases)** |  |  |  |
| **Coverage (1Q;median;mean;3Q)** | 30;37;37;44 | 155;254;320;402 | - |
| **Average no. of bases not covered** | 0 (0.0%) | 46 (<0.1%) | - |
| **Average no. of bases not covered with minimum read depth** | 1,648 (0.3%) | 10,070 (1.7%) | - |
| **WGS and HES (783,503bases)** |  |  |  |
| **Coverage (1Q;median;mean;3Q)** | 30;37;37;44 | - | 236;424;476;655 |
| **Average no. of bases not covered** | 1 (<0.1%) | - | 3,818 (0.5%) |
| **Average no. of bases not covered with minimum read depth** | 2,529 (0.3%) | - | 25,967 (3.3%) |
| **WES and HES (432,075bases)** |  |  |  |
| **Coverage (1Q;median;mean;3Q)** | - | 177;280;347;433 | 261;444;493;671 |
| **Average no. of bases not covered** | - | 17 (<0.1%) | 1,404 (0.3%) |
| **Average no. of bases not covered with minimum read depth** | - | 5,438 (1.3%) | 10,175 (2.4%) |
