# Supplementary material for: A comparative study of single nucleotide variant detection performance using three massively parallel sequencing methods
Source: PLoS One. 2020 Sep 28;15(9):e0239850. doi: 10.1371/journal.pone.0239850 (PMC7521702; doi:10.1371/journal.pone.0239850)
Supplement: S3 Table — (DOCX) [file pone.0239850.s003.docx]

**S3 Table. Fully exclusive (FE) and high quality fully exclusive (HQFE) variants from whole genome sequencing (WGS) and whole exome sequencing (WES) comparison.**

| **WGS and WES**  Size of investigated regions: 600,279bases | | | |
| --- | --- | --- | --- |
| **Number of:** | **WGS**  **(FE/HQFE)** |  | **WES  (FE/HQFE)** |
| WES variants present in WGS.bam with coverage ≥10 | - |  | 11/8 |
| WES variants present in WGS.bam with coverage ≤ 9 | - |  | 0/0 |
| WES variants not present in WGS.bam but genomic position has coverage ≥10 in WGS.bam | - |  | 1/0 |
| WES variants not present in WGS.bam but genomic position has coverage 1≤ 9 in WGS.bam | - |  | 0/0 |
| WES variant positions with zero coverage in WGS.bam | - |  | 0/0 |
| WGS variants present in WES.bam with coverage ≥40 | 45/32 |  | - |
| WGS variants present in WES.bam with coverage ≤ 39 | 8/5 |  | - |
| WGS variants not present in WES.bam but genomic position has coverage ≥40 in WES.bam | 10/0 |  | - |
| WGS variants not present in WES.bam but genomic position has coverage 1≤ 39 in WES.bam | 1/0 |  | - |
| WGS variant positions with zero coverage in WES.bam | 0/0 |  | - |
| **Total no. of variants:** | **64/38** |  | **12/8** |
| FE variants located within repetitive regions or regions difficult to sequence | 21  (33%) |  | 9 (75%) |
| HQFE variants located within repetitive regions or regions difficult to sequence | 11  (29%) |  | 6  (75%) |
| Hereof, HQFE variants located within GC-rich regions | 1  (3%) |  | 0  (0%) |
