# Supplementary material for: A comparative study of single nucleotide variant detection performance using three massively parallel sequencing methods
Source: PLoS One. 2020 Sep 28;15(9):e0239850. doi: 10.1371/journal.pone.0239850 (PMC7521702; doi:10.1371/journal.pone.0239850)
Supplement: S4 Table — (DOCX) [file pone.0239850.s004.docx]

**S4 Table. Fully exclusive (FE) and high quality fully exclusive (HQFE) variants from whole genome sequencing (WGS) and target enrichment sequencing (HES) comparison.**

| **WGS and HES**  Size of investigated regions: 783,503bases | | |
| --- | --- | --- |
| **Number of:** | **WGS**  **(FE/HQFE)** | **HES**  **(FE/HQFE)** |
| HES variants present in WGS.bam with coverage ≥10 | - | 146/104 |
| HES variants present in WGS.bam with coverage ≤ 9 | - | 26/17 |
| HES variants not present in WGS.bam but genomic position has coverage ≥10 in WGS.bam | - | 98/42 |
| HES variants not present in WGS.bam but genomic position has coverage 1≤ 9 in WGS.bam | - | 24/13 |
| HES variant positions with zero coverage in WGS.bam | - | 0/0 |
| WGS variants present in HES.bam with coverage ≥40 | 53/33 | - |
| WGS variants present in HES.bam with coverage ≤ 39 | 20/17 | - |
| WGS variants not present in HES.bam but genomic position has coverage ≥40 in HES.bam | 52/35 | - |
| WGS variants not present in HES.bam but genomic position has coverage 1≤ 39 in HES.bam | 17/12 | - |
| WGS variant positions with zero coverage in HES.bam | 45/44 | - |
| **Total no. of variants:** | **187/141** | **294/176** |
| FE variants located within repetitive regions or regions difficult to sequence | 55  (29%) | 194  (66%) |
| HQFE variants located within repetitive regions or or regions difficult to sequence | 33  (23%) | 131  (74%) |
| Hereof, HQFE variants located within GC rich regions | 25  (18%) | 9  (5%) |
