# Supplementary material for: A comparative study of single nucleotide variant detection performance using three massively parallel sequencing methods
Source: PLoS One. 2020 Sep 28;15(9):e0239850. doi: 10.1371/journal.pone.0239850 (PMC7521702; doi:10.1371/journal.pone.0239850)
Supplement: S5 Table — (DOCX) [file pone.0239850.s005.docx]

**S5 Table. Fully exclusive (FE) and high quality fully exclusive (HQFE) variants from whole exome sequencing (WES) and target enrichment sequencing (HES) comparison.**

| **WES and HES**  Size of investigated regions: 432.075 bases | | |
| --- | --- | --- |
| **Number of:** | **WES**  **(FE/HQFE)** | **HES**  **(FE/HQFE)** |
| HES variants present in WES.bam with coverage ≥40 | - | 23/5 |
| HES variants present in WES.bam with coverage ≤ 39 | - | 3/3 |
| HES variants not present in WES.bam but genomic position has coverage ≥40 in WES.bam | - | 4/0 |
| HES variants not present in WES.bam but genomic position has coverage 1≤ 39 in WES.bam | - | 4/0 |
| HES variant positions with zero coverage in WES.bam | - | 0/0 |
| WES variants present in HES.bam with coverage ≥40 | 9/8 | - |
| WES variants present in HES.bam with coverage ≤ 39 | 2/0 | - |
| WES variants not present in HES.bam but genomic position has coverage ≥40 in HES.bam | 1/0 | - |
| WES variants not present in HES.bam but genomic position has coverage 1≤ 39 in HES.bam | 2/1 | - |
| WES variant positions with zero coverage in HES.bam | 8/3 | - |
| **Total no. of variants:** | **22/12** | **34/8** |
| FE variants located within repetitive regions or regions difficult to sequence | 13  (59%) | 15  (44%) |
| HQFE variants located within repetitive regions or regions difficult to sequence | 4  (33%) | 2  (25%) |
| Hereof, HQFE variants located within GC-rich regions | 3  (25%) | 0  (0%) |
