# Supplementary material for: A comparative study of single nucleotide variant detection performance using three massively parallel sequencing methods
Source: PLoS One. 2020 Sep 28;15(9):e0239850. doi: 10.1371/journal.pone.0239850 (PMC7521702; doi:10.1371/journal.pone.0239850)
Supplement: S6 Table — WGS: Whole genome sequencing, WES: whole exome sequencing, and HES: Haloplex target enrichment system. 1/1 shows a homozygote variant genotype and 0/1 shows a heterozygote genotype. SNVs in italic are found in the regions sequenced by all methods. (DOCX) [file pone.0239850.s006.docx]

**S6 Table. Single nucleotide variants (SNVs) with discordant genotypes.** WGS: Whole genome sequencing, WES: whole exome sequencing, and HES: Haloplex target enrichment system. 1/1 shows a homozygote variant genotype and 0/1 shows a heterozygote genotype. SNVs in italic are found in the regions sequenced by all methods.

| **WES vs WGS** | | | |
| --- | --- | --- | --- |
| **rsID** | **Genotype WES** | **Genotype WGS** | **Sample** |
| *rs185169879* | *1/1* | *0/1* | *Sample8* |
| rs2074238 | 1/1 | 0/1 | Sample8 |
| rs3778731 | 0/1 | 1/1 | Sample7 |
| rs56177217 | 1/1 | 0/1 | Sample3 |
| rs56177217 | 1/1 | 0/1 | Sample8 |
| rs56177217 | 1/1 | 0/1 | Sample10 |
| **WES vs HES** | | | |
| **rsID** | **Genotype WES** | **Genotype HES** | **Sample** |
| *rs1269041* | *0/1* | *1/1* | *Sample7* |
| *rs17277970* | *0/1* | *1/1* | *Sample2* |
| *rs185169879* | *1/1* | *0/1* | *Sample8* |
| *rs2277474* | *0/1* | *1/1* | *Sample10* |
| *rs7268512* | *0/1* | *1/1* | *Sample7* |
| **WGS vs HES** | | | |
| **rsID** | **Genotype WGS** | **Genotype HES** | **Sample** |
| rs1043802 | 0/1 | 1/1 | Sample1 |
| rs1043802 | 0/1 | 1/1 | Sample2 |
| rs1043802 | 0/1 | 1/1 | Sample10 |
| rs12406863 | 1/1 | 0/1 | Sample3 |
| *rs1269041* | *0/1* | *1/1* | *Sample7* |
| rs1272944 | 1/1 | 0/1 | Sample1 |
| rs1272944 | 1/1 | 0/1 | Sample3 |
| rs1272944 | 1/1 | 0/1 | Sample7 |
| rs1272944 | 1/1 | 0/1 | Sample8 |
| rs12758915 | 0/1 | 1/1 | Sample2 |
| rs1537406 | 0/1 | 1/1 | Sample7 |
| rs1723484 | 0/1 | 1/1 | Sample7 |
| *rs17277970* | *0/1* | *1/1* | *Sample2* |
| rs2269350 | 0/1 | 1/1 | Sample10 |
| *rs2277474* | *0/1* | *1/1* | *Sample10* |
| rs2846694 | 0/1 | 1/1 | Sample2 |
| rs2846694 | 0/1 | 1/1 | Sample3 |
| rs2846694 | 0/1 | 1/1 | Sample8 |
| rs4748484 | 1/1 | 0/1 | Sample2 |
| rs4748485 | 1/1 | 0/1 | Sample2 |
| rs593288 | 0/1 | 1/1 | Sample4 |
| *rs7268512* | *0/1* | *1/1* | *Sample7* |
|  | | | |
